# Urinary angiostatin, CXCL4 and VCAM-1 as biomarkers of lupus nephritis

**DOI:** 10.1186/s13075-017-1498-3

**Published:** 2018-01-11

**Authors:** Chi Chiu Mok, Samar Soliman, Ling Yin Ho, Fatma A. Mohamed, Faten Ismail Mohamed, Chandra Mohan

**Affiliations:** 1 Department of Medicine, Tuen Mun Hospital, Tsing Chung Koon Road, New Territories, Hong Kong, China; 2 Rheumatology and Rehabilitation Department, Faculty of Medicine, Minya University, Minya, Egypt; 3 Department of Biomedical Engineering, Houston, TX USA

**Keywords:** Biomarker, Lupus, Nephritis, Adhesion molecule, Anti-angiogenic, Chemokine

## Abstract

**Background:**

The aim was to study urinary angiostatin, CXC chemokine ligand 4 (CXCL4) and vascular cell adhesion molecule-1 (VCAM-1) as biomarkers of renal disease in systemic lupus erythematosus (SLE).

**Method:**

Patients who fulfilled ≥ 4 American College of Rheumatology (ACR) criteria for SLE with active renal, active non-renal or inactive disease, and a group of healthy controls were studied. Urine samples were assayed for angiostatin, CXCL4 and VCAM-1 by ELISA, and normalized by creatinine. Receiver operating characteristic analysis was performed to obtain the best cutoff values to calculate the performance of these markers in differentiating the different groups of patients as compared to anti-double-stranded DNA (anti-dsDNA) and complement C3. Correlation between these urinary biomarkers and various renal parameters was also tested.

**Results:**

Patients with SLE (n = 227; 80 with inactive SLE, 67 with active non-renal disease and 80 with active renal disease; 94% women; age 39.2 ± 13.8 years) and 53 controls (96% women) were studied. All were ethnic Chinese. Urinary angiostatin, CXCL4 and VCAM-1 (normalized for creatinine) were significantly higher in patients with active renal disease than in patients with active non-renal disease, patients with inactive SLE and controls. These markers correlated significantly with total SLE disease activity index (SLEDAI) and renal SLEDAI scores, and with the urinary protein-to-creatinine ratio. Urine angiostatin exhibited higher specificity and sensitivity in differentiating active renal from active non-renal SLE (area under the curve (AUC) 0.87) than serum anti-dsDNA/C3. Urine CXCL4 (AUC 0.64) and VCAM-1 (AUC 0.73), on the other hand, performed similarly to anti-dsDNA/C3. All three markers performed comparably to anti-dsDNA/C3 in distinguishing active from inactive SLE. In a subgroup of 68 patients with paired renal biopsy, the urinary levels of these proteins did not differ significantly between the proliferative and non-proliferative types of lupus nephritis. Urinary CXCL4 and VCAM-1 correlated significantly with the histologic activity score, and urinary angiostatin correlated significantly with proteinuria in this subgroup.

**Conclusions:**

Urinary angiostatin, CXCL4 and VCAM-1 are potential biomarkers for SLE, in particular lupus nephritis. Further longitudinal studies are necessary to delineate the performance of these markers in predicting renal flares and prognosis in SLE patients.

## Background

Systemic lupus erythematosus (SLE) is a complex systemic autoimmune disease with unknown etiology [1]. Among the various clinical manifestations of SLE, renal involvement is one of the most important causes of morbidity and mortality. Lupus renal disease is more prevalent and severe in certain ethnic groups such as African Americans, Hispanics and Asians [2]. Mortality in SLE increases at least 1.2-fold when the kidney is affected [3], and 10-year cumulative renal survival in patients with proliferative lupus nephritis (LN) ranges from 74 to 81% [4, 5]. Moreover, glomerulonephritis in patients with SLE significantly reduces their quality of life [6] and working ability [7].

Current laboratory markers for LN such as proteinuria, creatinine clearance, anti-double-stranded DNA (anti-dsDNA), and complement levels are not ideal. They lack sensitivity and specificity for distinguishing renal inflammation and damage, or predicting flare of nephritis [8]. Ongoing renal activity may not be detected by conventional markers. For instance, chronic lesions may contribute to persistent proteinuria rather than active renal inflammation. At present, renal biopsy is the gold standard for assessing histological severity and chronic lesions in LN. However, it is invasive and serial biopsies are impractical in monitoring treatment of LN. Therefore, novel biomarkers for early diagnosis of renal disease and prediction of kidney flares in SLE have to be explored.

Over the past decade, a myriad of novel biomarkers have been studied in LN [2, 8–10]. Urinary biomarkers are attractive candidates for tracking LN activity as they are directly excreted from the kidneys and readily available for examination [8]. However, to date, no biomarkers have been adequately validated for routine clinical use in patients with LN.

An unbiased, high-throughput proteomics approach enables simultaneous evaluation of a large number of proteins in an efficient manner. Recent proteomic studies from our group [11, 12] and others [13] have identified urinary angiostatin, vascular cell adhesion molecule-1 (VCAM-1) and CXC chemokine ligand 4 (CXCL4) as potential urinary biomarkers of LN. Angiostatin is a proteolytic fragment of plasminogen that has been found to inhibit angiogenesis in cancer [14]. Our previous analysis revealed increased levels of urinary angiostatin in patients with active SLE, particularly those with diffuse proliferative LN [11]. Urinary angiostatin differentiates patients with active SLE from those with inactive SLE, and correlated significantly with SLE activity and the renal pathology chronicity index [11]. VCAM-1 is an adhesion molecule involved in trafficking of inflammatory cells and lymphocytes. Serum and urine VCAM-1 has been shown to be elevated in patients with active SLE or LN [15–20]. Our previous study has shown that urinary VCAM-1 level is elevated in patients with SLE compared to controls, and is correlated with renal activity and SLEDAI scores [20]. CXCL4, also known as platelet factor 4 (PF4), is a potent anti-angiogenic chemokine [21]. A recent proteome-wide analysis showed that circulating CXCL4 is elevated in patients with systemic sclerosis, and is correlated with the risk of progression of skin and lung fibrosis and pulmonary hypertension [22]. However, there are still no data on CXCL4 in LN.

In view of the paucity of data on these three urinary protein markers in LN, particularly in Chinese patients, we conducted this cross-sectional study to evaluate the performance of these markers in predicting active renal disease in SLE, as compared to conventional SLE markers.

## Methods

### Study population

Adult patients (≥18 years of age) diagnosed as having SLE according to the 1997 American College of Rheumatology (ACR) classification criteria [23] were recruited from our Rheumatology outpatient clinics or when they were hospitalized in our unit between August 2012 and June 2015. Blood was taken for assessment of SLE activity (anti-dsDNA, complement C3/4 level) and urine samples were collected for the assay of the three biomarkers studied, namely angiostatin, CXCL4 and VCAM-1. Blood and urine samples were collected from those with active SLE before augmentation of immunosuppressive therapies. A group of healthy subjects were also recruited as controls. Written informed consent was obtained from the participants and this study was approved by the Ethics Committee of our hospital administration.

Patients recruited were stratified into three groups: clinically inactive SLE, active non-renal SLE and active renal SLE. The fourth group comprised healthy controls. Clinical data that include demographic and clinical characteristics, renal parameters (histological classes of LN, urine protein-to-creatinine ratio and sediments, and serum creatinine in those with active renal disease) were collected at the time of recruitment. SLE disease activity and organ damage was assessed. Urinary protein marker levels were compared in these patient groups and controls. Correlation between the urinary markers and various renal parameters was also tested.

### Assessment of disease activity and organ damage

SLE disease activity was assessed by the Safety of Estrogens in Lupus Erythematosus National Assessment (SELENA) version of the SLEDAI (SELENA–SLEDAI), which is a validated tool to assess lupus activity in the multicenter randomized controlled SELENA trial for the safety of estrogen use in patients with SLE [24, 25]. “Clinically inactive SLE” included patients with total clinical SLEDAI = 0 and no clinical activity in other systems that are not captured by the SLEDAI. “Active renal SLE” was defined as patients with renal SLEDAI ≥ 4, while “active non-renal SLE” included patients with total clinical SLEDAI ≥1 and/or clinical activity in other systems not captured by the SLEDAI, but excluding patients with “active renal SLE”.

The physician’s global assessment (PGA) of disease activity of SLE (range 0–3) [26] was also performed by the attending rheumatologists to grade their impression of the patient’s disease activity at the time of venipuncture.

### Assay of serum and urinary protein markers

Serologic titers of anti-dsDNA were measured by commercial ELISA (Euro Diagnostica). Serum complement levels were assayed by Immunoturbidimetry (Abbott Architect). Urinary levels of angiostatin, CXCL4 and VCAM1 were assayed using ELISA. In particular, CXCL4 (catalog number DY795) and VCAM1 (catalog number DY809) were assayed using ELISA kits from R&D Systems (Minneapolis, MN, USA), whereas angiostatin was assayed using an ELISA kit (catalog number ELH-Angiostatin) from Raybiotech, Inc (Norcross, GA, USA). Urine samples were diluted 1: 5 for CXCL4, 1:100 for VCAM1 and 1:2 for angiostatin. Optical densities at 450 nm were measured using a microplate reader ELX808 (BioTek Instruments, Winooski, VT, USA) and sample concentrations were calculated using a standard curve. All measurements were assayed in duplicate. The values of these urinary protein markers were normalized to urine creatinine.

### Statistical analyses

Unless otherwise stated, values in this study were expressed as mean ± standard deviation (SD). Comparison of values among different groups of subjects was performed using the non-parametric Kruskal-Wallis H (continuous variables) and chi-square (categorical variables) tests. Correlation analysis between two variables was performed using Spearman’s rank correlation. Receiver operating characteristic (ROC) curve analysis was employed to study the best cutoff values of the protein markers to differentiate between active renal and non-renal SLE and between active and inactive SLE. The area under the curve (AUC) was calculated and the best trade-off point of sensitivity and specificity was determined from the values calculated for each of the coordinates on the curve.

Elevation of the protein markers was defined using the best cutoff values obtained from ROC analyses. The sensitivity, specificity, positive predictive value (PPV) and negative predictive value (NPV) of each of the markers was calculated using 2 × 2 contingency tables. Sensitivity was calculated as the ratio of true positive (TP) to TP plus false negative (FN). Specificity was equal to true negative (TN) divided by the sum of TN and false positive (FP). The PPV was calculated by the ratio of TP to TP plus FP and finally, the NPV was the ratio of TN to the sum of FN and TN.

Statistical significance was defined as a two-tailed *P* value less than 0.05. All statistical analysis was performed using SPSS (version 16.0, Chicago, IL, USA).

## Results

### Study population

A total of 227 patients with SLE (94% women) were studied. The mean age was 39.2 ± 13.8 years and mean SLE duration was 7.3 ± 7.0 years. All were ethnic Chinese. There were 80 patients (35%) with active renal SLE, 67 (30%) with active non-renal SLE and 80 (35%) with clinically inactive SLE. Fifty-three healthy subjects (96% women; mean age 25.8 ± 3.9 years) were recruited as controls.

Table 1 shows the clinical characteristics of the patients with SLE in the study. Patients with inactive SLE were significantly older and had longer SLE duration than the other patients. Patients with active renal disease were more likely to have anti-La antibody but less likely to be positive for antiphospholipid antibodies. The total SLEDAI and PGA scores were significantly higher in patients with active renal than with non-renal SLE. Mycophenolate mofetil and tacrolimus was more frequently used in patients with active renal SLE, whereas hydroxychloroquine was more often used in patients with active non-renal SLE. The Systemic Lupus International Collaborating Clinics (SLICC) organ damage scores, however, were similar among the three groups of patients with SLE.

**Table 1:** Clinical characteristics of the patients with SLE in the study

	Inactive SLE (*N* = 80)	Active non-renal SLE (*N* = 67)	Active renal SLE (*N* = 80)	Total (*N* = 227)	*P* value*
	Mean ± SD; number (%)				
Age, years	44.0 ± 12.4	35.1 ± 13.9	37.8 ± 13.9	39.2 ± 13.8	< 0.001
Women	77 (96)	62 (93)	75 (94)	214 (94)	0.37
SLE duration, years	12.5 ± 6.6	4.0 ± 5.2	4.9 ± 5.8	7.3 ± 7.0	< 0.001
Clinical disease activity					
Neuropsychiatric	-	9 (13)	5 (6.3)	14 (6.2)	0.14
Musculoskeletal	-	30 (45)	29 (36)	59 (26)	0.29
Renal	-	0 (0)	80 (100)	80 (35)	< 0.001
Mucocutaneous	-	37 (55)	26 (33)	63 (28)	0.006
Serositis	-	15 (22)	14 (18)	29 (13)	0.46
Hematological	-	39 (58)	19 (24)	58 (26)	< 0.001
Autoantibodies					
Anti-Sm	12 (15)	21 (31)	21 (26)	54 (24)	0.06
Anti-Ro	48 (60)	44 (66)	57 (71)	149 (66)	0.37
Anti-La	11 (14)	15 (22)	27 (34)	53 (23)	0.01
Anti-nRNP	20 (25)	28 (42)	30 (38)	78 (34)	0.09
aPL^a^	31 (39)	27 (40)	18 (23)	76 (33)	0.04
SLEDAI	2.11 ± 1.67	10.1 ± 5.51	16.0 ± 6.42	9.39 ± 7.65	< 0.001
PGA score	0.21 ± 0.15	1.89 ± 0.32	2.14 ± 0.21	1.39 ± 0.90	< 0.001
SLICC damage score	0.49 ± 1.20	0.69 ± 0.97	0.81 ± 1.32	0.77 ± 1.18	0.98
Medications at time of sample collection					
Prednisolone	38 (48)	27 (40)	41 (51)	106 (47)	0.41
Azathioprine	30 (38)	8 (12)	16 (20)	54 (24)	0.001
Cyclophosphamide	0 (0)	1 (1.5)	1 (1.3)	2 (0.9)	0.57
Cyclosporin A	3 (3.8)	2 (3)	3 (3.8)	8 (3.5)	0.96
Tacrolimus	2 (2.5)	3 (4.5)	2 (2.5)	7 (3)	0.73
Mycophenolate mofetil	12 (15)	6 (9)	11 (14)	29 (13)	0.52
Hydroxychloroquine	48 (60)	33 (49)	35 (44)	116 (51)	0.11

*SLE* systemic lupus erythematosus, *SD* standard deviation, *SLEDAI* SLE disease activity index, *aPL* antiphospholipid, *PGA* physician’s global assessment, *SLICC* SLE International Collaborative Clinic

*P comparison among the three groups

^a^Either IgG anti-cardiolipin or the lupus anticoagulant

### Urine levels of angiostatin, CXCL4 and VCAM-1

**Fig. 1:**
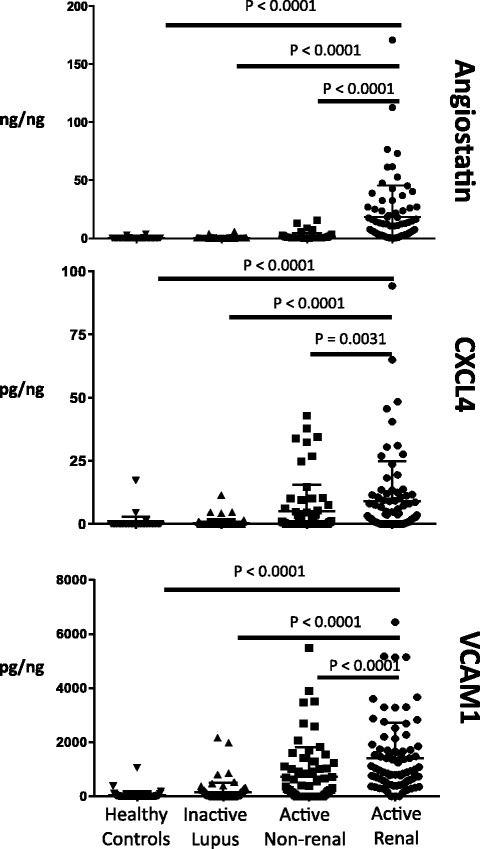
Urinary levels of angiostatin, CXC chemokine ligand 4 (CXCL4) and vascular cell adhesion molecule-1 (VCAM-1) in the four groups of subjects studied. Values were normalized to urinary creatinine. The lower horizontal line of the error bar represents the mean and the upper horizontal line is mean + one standard deviation

Figure 1 shows the urine levels of angiostatin, CXCL4 and VCAM-1 in the four groups of subjects studied. Levels of all three protein markers were significantly higher in patients with active renal disease than in those with active non-renal disease or inactive SLE and healthy controls (Table 2). Among patients with inactive SLE (N = 80), urinary levels of angiostatin (0.38 ± 0.65 vs 0.31 ± 0.48 ng/ng; *p* = 0.49), CXCL4 (0.46 ± 1.97 vs 0.31 ± 1.01 pg/ng; *p* = 0.59) and VCAM-1 (169 ± 380 vs 150 ± 337 pg/ng; p = 0.63) were slightly higher in those who had a history of renal disease (N = 39) than in those who did not (N = 41). However, the differences were not statistically significant.

**Table 2:** Urine protein markers in the subjects studied

Urine protein markers (normalized to creatinine)	Healthy controls (*N* = 53)	Inactive SLE (*N* = 80)	Active non-renal SLE (*N* = 67)	Active renal SLE (*N* = 80)	*P* value
Angiostatin (ng/ng)	0.26 ± 0.60	0.34 ± 0.56	1.60 ± 2.91	18.4 ± 27.1	< 0.0001^a^ < 0.0001^b^
CXCL4 (pg/ng)	0.11 ± 0.63	0.38 ± 1.55	5.12 ± 10.4	9.11 ± 15.7	0.0031^a^ < 0.0001^b^
VCAM-1 (pg/ng)	18.5 ± 60.8	159 ± 357	722 ± 1100	1410 ± 1310	< 0.0001^a^ < 0.0001^b^

Values are expressed as mean ± standard deviation

*SLE* systemic lupus erythematosus*, CXCL4* CXC chemokine ligand 4, *VCAM-1* vascular cell adhesion molecule-1

^a^*P* value for active renal vs active non-renal groups

^b^*P* value for active SLE vs inactive SLE groups

**Fig. 2:**
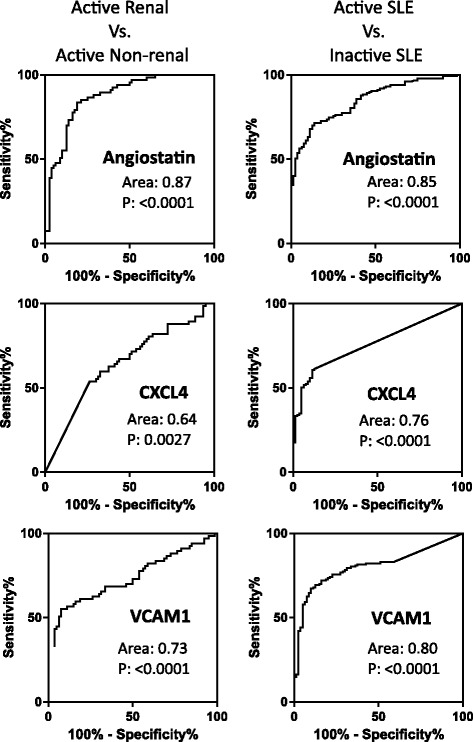
Receiver operating characteristic curves of the three protein markers in differentiating active renal from active non-renal systemic lupus erythematosus (SLE) and active SLE from inactive SLE. *CXCL4* CXC chemokine ligand 4, *VCAM-1* vascular cell adhesion molecule-1

ROC curve analyses were performed to derive the best cutoff values of these protein markers to differentiate between active renal and non-renal SLE and between active SLE and inactive SLE (Fig. 2). The AUCs and best cutoff values are shown in Table 3. Among the three urine protein markers, angiostatin exhibited the highest AUC and specificity/sensitivity in differentiating active renal from active non-renal SLE. CXCL4 and VCAM-1, on the other hand, had a similar AUC and specificity/sensitivity to conventional serological markers (anti-dsDNA and complement C3) in distinguishing between active renal and non-renal SLE. All three urine protein markers had a similar AUC and specificity/sensitivity metrics in differentiating active SLE from inactive SLE compared to the conventional markers, serum anti-dsDNA and C3 levels.

**Table 3:** ROC analyses of the cutoff values of the protein markers

Markers	Cutoff	AUC (95% CI)	Specificity	Sensitivity	*P* value
Active renal vs active non-renal					
Angiostatin	> 2.219 ng/ng	0.87 (0.81–0.92)	0.82	0.80	< 0.001
CXCL4	> 1.06 pg/ng	0.64 (0.55–0.73)	0.61	0.63	0.003
VCAM-1	> 668 pg/ng	0.73 (0.65–0.82)	0.66	0.69	< 0.001
Anti-dsDNA	> 208 IU/ml	0.64 (0.55–0.73)	0.64	0.63	0.004
C3	< 0.50 g/L	0.66 (0.58–0.75)	0.61	0.66	0.001
Active vs inactive SLE					
Angiostatin	> 0.345 ng/ng	0.85 (0.81–0.90)	0.71	0.78	< 0.001
CXCL4	> 0.085 pg/ng	0.76 (0.70–0.82)	0.88	0.61	< 0.001
VCAM-1	> 179 pg/ng	0.80 (0.75–0.86)	0.73	0.76	< 0.001
Anti-dsDNA	> 159 IU/ml	0.75 (0.69–0.81)	0.65	0.65	< 0.001
C3	< 0.72 g/L	0.82 (0.76–0.87)	0.73	0.74	< 0.001

*ROC* receiver operating characteristic curve, *AUC* area under the curve, *SLE* systemic lupus erythematosus, *CI* confidence interval, *CXCL4* CXC chemokine ligand 4, *VCAM-1* vascular cell adhesion molecule-1

Table 4 shows the performance of the three urine protein markers in discriminating active renal from active non-renal disease, and active SLE from inactive SLE in comparison with serum anti-dsDNA and low complement C3 level. Again, urine angiostatin showed a higher specificity, sensitivity, positive and negative predictive values in differentiating active renal from active non-renal disease than CXCL4 or VCAM-1. The latter two markers exhibited similar performance to anti-dsDNA or low C3 in differentiating the different groups of Patients with SLE.

**Table 4:** Performance of the protein markers in differentiating different groups of patients with SLE

Markers	Sensitivity	Specificity	PPV	NPV
Active renal vs active non-renal				
Angiostatin	0.80	0.82	0.84	0.77
CXCL4	0.63	0.61	0.66	0.58
VCAM-1	0.69	0.66	0.71	0.64
Anti-dsDNA	0.63	0.64	0.68	0.59
Low C3	0.61	0.66	0.68	0.59
Active vs inactive SLE				
Angiostatin	0.78	0.71	0.83	0.63
CXCL4	0.61	0.88	0.90	0.55
VCAM-1	0.76	0.73	0.83	0.62
Anti-dsDNA	0.65	0.65	0.78	0.51
C3	0.74	0.73	0.84	0.61

*PPV* positive predictive value, *NPV* negative predictive value, *SLE* systemic lupus erythematosus, *CXCL4* CXC chemokine ligand 4, *VCAM-1* vascular cell adhesion molecule-1

### Correlation between urine protein markers and SLE activity and renal parameters

Among patients with SLE (*N* = 227), the three urine protein markers correlated significantly with the total SLEDAI (angiostatin, Rho 0.60, *p* < 0.001; CXCL4, Rho 0.46, *p* < 0.001; VCAM-1, Rho 0.53, *p* < 0.001), renal SLEDAI (angiostatin, Rho 0.66, *p* < 0.001; CXCL4, Rho 0.45, *p* < 0.001; VCAM-1: Rho 0.51, *p* < 0.001) and PGA score (angiostatin: Rho 0.54, *p* < 0.001; CXCL4, Rho 0.45, *p* < 0.001; VCAM-1, Rho 0.56, *p* < 0.001). These markers also correlated significantly with the urine protein-to-creatinine ratio (uP/Cr) (angiostatin, Rho 0.73, *p* < 0.001; CXCL4, Rho 0.51, *p* < 0.001; VCAM-1, Rho 0.59, *p* < 0.001).

A subgroup of 73 patients with active nephritis in whom renal biopsy was performed was further studied. Five patients were excluded because the interval between urine sample collection and renal biopsy exceeded 6 weeks. In the remaining 68 patients (in whom the mean interval between urine sample collection and renal biopsy was 1.1 ± 1.4 weeks), urine levels of the three protein markers did not differ significantly between those with proliferative types of lupus nephritis (International Society of Nephrology/Renal Pathology Society (ISN/RPS) class III/IV ± V; N = 50) and other histological classes of nephritis (ISN/RPS I/II/pure V; N = 18) (Table 5). In these biopsy-concurrent urine samples, urine CXCL4 (Rho 0.25, *p* = 0.049) and VCAM-1 (Rho −0.28, *p* = 0.02), but not angiostatin (Rho 0.11, *p* = 0.39), correlated significantly with the histologic activity index. However, there was no significant association between the three urine protein markers and the histologic chronicity index or renal SLEDAI score. On the other hand, urine angiostatin levels (Rho 0.36, *p* = 0.003), but not CXCL4 (Rho 0.07, *p* = 0.59) or VCAM-1 (Rho −0.11, *p* = 0.36), correlated significantly with the uP/Cr ratio in this subgroup of patients.

**Table 5:** Urine protein markers in 68 patients with active lupus nephritis confirmed by renal biopsy

	ISN/RPS class III/IV ± V lupus nephritis (*N* = 50)	ISN/RPS class I, II, V lupus nephritis (*N* = 18)	*P* value
Urinary angiostatin (ng/ng)	19.0 ± 27.8	17.6 ± 23.0	0.83
Urinary CXCL4 (pg/ng)	9.95 ± 16.7	4.22 ± 7.31	0.06
Urinary VCAM-1 (pg/ng)	1330 ± 1380	1560 ± 1140	0.50
Serum anti-dsDNA (IU/ml)	254 ± 81.2	179 ± 116	0.02
Serum C3 (g/L)	0.43 ± 0.17	0.60 ± 0.26	0.02
Histological activity index	8.9 ± 3.2	1.6 ± 2.0	< 0.001
Histological chronicity index	2.5 ± 0.8	0.9 ± 1.3	0.001
Renal SLEDAI score	9.3 ± 3.1	6.4 ± 2.4	< 0.001
Urinary P/Cr	3.3 ± 2.3	4.1 ± 5.4	0.56

*SLEDAI* systemic lupus erythematosus disease activity index, *CXCL4* CXC chemokine ligand 4, *VCAM-1* vascular cell adhesion molecule-1*P/Cr* protein-to-creatinine ratio, *ISN/RPS* International Society of Nephrology/Renal Pathology Society

## Discussion

In this cross-sectional study, we showed that the urinary levels of angiostatin, CXCL4 and VCAM-1 were significantly higher in patients with active renal SLE than in patients with active non-renal or inactive SLE. The urinary levels of these markers correlated significantly with the SLE disease activity score, renal activity scores and urinary protein levels. These markers were able to differentiate active renal from active non-renal SLE, and active from inactive SLE. Among patients with biopsy-proven active LN, urinary CXCL4 and VCAM-1 correlated with biopsy activity index but not proteinuria. On the other hand, urine angiostatin correlated with proteinuria but not the biopsy activity index. These observations suggest that the pathogenic mechanisms that lead to proteinuria and the histological changes that contribute to renal pathology “activity” may not be the same. As an example, podocyte loss may contribute to proteinuria but not a change in the activity index. Further studies are warranted to investigate how angiostatin is related mechanistically to proteinuria, and to fathom which specific aspects of renal pathology “activity” are impacted by CXCL4 and VCAM-1.

Angiostatin, the N-terminal fragment of plasminogen, is a potent angiogenesis inhibitor that has been shown to mediate suppression of metastases from Lewis lung carcinoma [27]. Angiostatin specifically inhibits proliferation and induces apoptosis of the vascular endothelial cells, thus inhibiting tumor growth [28]. More recently, angiostatin has been shown to have anti-inflammatory properties by inhibition of activation and migration of neutrophils [29]. In a mouse model of chronic kidney injury, treatment with recombinant adeno-associated viruses expressing angiostatin was shown to retard the progression of kidney disease, likely due to the anti-inflammatory actions of this anti-angiogenic protein [30]. In our previous study of African American, Hispanic and Caucasian patients with SLE, urinary angiostatin was increased in active SLE, particularly in active LN [11]. Urinary angiostatin correlated significantly with renal SLEDAI and the histologic chronicity index. The results of the present study, which involved a larger group of Chinese patients with SLE, confirmed the finding that urinary angiostatin is a marker that can differentiate active renal from active non-renal SLE with a higher specificity/sensitivity than anti-dsDNA and complement C3. Although urinary angiostatin correlated with the degree of proteinuria, we were unable to show that angiostatin correlated with the renal histologic severity or activity in a subgroup analysis, probably related to the limited sample of patients with different histological classes of LN and a considerable proportion of patients having mixed histological classes of LN.

VCAM-1, a member of the immunoglobulin superfamily, is an adhesion molecule involved in the recruitment of inflammatory cells via interaction with an integrin located on leukocytes [31]. Soluble VCAM-1 levels are elevated in several autoimmune diseases that include SLE and rheumatoid arthritis [15–17, 32]. Previous studies have demonstrated that urinary VCAM-1 is elevated in patients with active SLE or LN [18–20]. We have previously shown that urinary VCAM-1 is increased in African Americans, Hispanic and Caucasian patients with LN, and correlates with SLEDAI scores and histologic renal activity [20]. This is consistent with the current study in Chinese patients showing that urinary VCAM-1 levels were elevated in active LN, and differentiated active renal from non-renal disease. As noted above, urinary VCAM-1 did not correlate with the degree of proteinuria but correlated significantly (but negatively) with the histologic activity index. The reciprocal relationship between urinary VCAM-1 and histologic activity is intriguing. One plausible explanation was that in our study, there were higher urinary levels of VCAM-1 in patients with pure membranous LN, who had significantly lower histological activity (data not shown). This may contribute to the paradoxical negative relationship between VCAM-1 level and histologic activity score. As urine VCAM-1 did not correlate with the degree of proteinuria, it should be further explored as a urinary marker that may predict flares of LN independent of proteinuria, or preceding proteinuria.

Similar to angiostatin, CXCL4 is another potent anti-angiogenic chemokine that influences angiogenesis by an integrin-dependent mechanism [21]. Circulating CXCL4 levels are increased in patients with systemic sclerosis and correlate with progression of heart and lung disease [22]. CXCL4 downregulates the expression of the anti-fibrotic cytokine-like interferon-γ but upregulates pro-fibrotic cytokines such as IL-4 and IL-13 [33]. It also promotes proliferation of the regulatory T cells while impairing their function, which may play a role in the regulation of the immune system [34]. Because the platelet is the main source of circulating CXCL4, this chemokine is postulated to be associated with atherosclerosis and thrombosis [35, 36]. More recent in vitro and murine data also suggest that the plasmacytoid dendritic cells are also capable of producing CXCL4 [22]. However, the origin and mechanism of CXCL4 excretion in the urine in patients with immune-mediated glomerulonephritis remains unclear. A study of patients with subclinical tubulitis, which was associated with the development of chronic kidney tubular lesions, did not report elevation of urinary CXCL4 [37]. In the current study, urinary CXCL4 was elevated in patients with active SLE and LN. Although the discriminating power of CXCL4 for active renal disease was not superior to angiostatin, it correlated significantly with the histologic activity scores on renal biopsy. CXCL4 should further be evaluated as a potential biomarker for LN flares and prognosis (renal fibrosis) in long-term longitudinal studies.

There are several limitations of the current study. First, the design is cross-sectional. Although we showed that these novel urinary markers correlated with SLE renal activity and differentiated active renal from non-renal SLE, their role in predicting flares and progression of LN is still unclear. This has to be addressed through long-term longitudinal studies. Moreover, whether these markers are more sensitive than urinary sediments in the detection of renal activity is unknown, as data on urinary sediments in patients with inactive SLE or active non-renal SLE were unavailable for analyses. Second, renal histologic assessment in the 68 patients with LN was performed by different pathologists and there might be inter-observer variation in the assessment. This, coupled with the relatively small sample of each histological class of LN, might have contributed to the negative correlation between renal histological classes and urine marker levels in some instances. Although immunohistochemical analysis of the biopsy tissues was not performed in this study, we have previously demonstrated expression of VCAM-1 and angiostatin in kidney tissue from humans and mice with LN [11, 38]. Renal CXCL4 expression studies of are in progress.

Despite these caveats, our study has provided further evidence to suggest a potential role of urinary angiostatin, CXCL4 and VCAM-1 as predictors of renal involvement in patients with SLE. Because of the shortcomings of existing clinical serological and renal parameters in the monitoring and prognostic stratification of LN, the quest for novel biomarkers has to be continued. Further prospective studies will provide more information on the performance of these urinary protein markers in predicting flares and prognosis of LN as compared to conventional markers and urinary protein quantification.

## Conclusion

In this study, we showed that the novel urinary biomarkers angiostatin, CXCL4 and VCAM-1 differentiate active renal from active non-renal disease in patients with SLE. The urinary levels of these biomarkers correlated significantly with SLEDAI, renal SLEDAI and urine protein levels. Among patients with biopsy-proven active LN, urinary CXCL4 and VCAM-1 correlated significantly with the histologic activity index but not proteinuria. On the other hand, urinary angiostatin correlated with proteinuria but not the biopsy activity index. These data suggest the mechanisms of proteinuria and histological activity may not necessarily be the same in LN. Longitudinal studies are needed to evaluate the performance of these urinary markers in predicting flares and prognosis of LN as compared to conventional markers and urinary protein quantification.

## Abbreviations

ACR: American College of Rheumatology
AUC: Area under the curve
CXCL4: CXC chemokine ligand 4
dsDNA: Double-stranded DNA
ELISA: Enzyme-linked immunosorbent assay
FN: False negative
FP: False positive
IL13: Interleukin-13
IL4: Interleukin-4
ISN/RPS: International Society of Nephrology/Renal Pathology Society
LN: Lupus nephritis
NPV: Negative predictive value
PGA: Physician’s global assessment
PPV: Positive predictive value
ROC: Receiver operating characteristic
SD: Standard deviation
SELENA: Safety of Estrogens in Lupus Erythematosus National Assessment
SLE: Systemic lupus erythematosus
SLEDAI: Systemic lupus erythematosus disease activity index
SLICC: Systemic Lupus International Collaborating Clinics
TP: True positive
uP/Cr: Urine protein-to-creatinine ratio
VCAM-1: Vascular cell adhesion molecule-1
